# A bifurcation analysis of a modified neural field model: conductance-based synapses act as an anti-epileptic regulatory mechanism

**DOI:** 10.1186/1471-2202-12-S1-P24

**Published:** 2011-07-18

**Authors:** Andre DH Peterson, Iven MY Mareels, Hamish Meffin, David B Grayden, Mark J Cook, Anthony N Burkitt

**Affiliations:** 1 University of Melbourne, Victoria 3010, Australia; 2 The Bionic Ear Institute, East Melbourne, Victoria 3002, Australia; 3 St. Vincent’s Hospital Melbourne, Victoria 3065, Australia; 4 NICTA Victoria Research Laboratory, Victoria 3010, Australia

## 

The spread of seizure-like behaviour through the cortex is facilitated not only by hyper-excitable, hyper-synchronous neuronal population firing, but by overcoming the regulatory mechanisms of the brain, such as feedback and feed-forward inhibition. These control mechanisms normally stabilise such pathological behaviour [1]. We suggest an additional network regulatory mechanism in the form of a ‘shunting’ effect based on the properties of conductance-based synapses, an important neurophysiological structure whose mechanism is often overlooked in macroscopic models of brain dynamics.

A mathematical neural field model [2] is modified to include conductance-based synapses as opposed to current-based synapses. This is a more realistic description of synaptic dynamics that has a significant effect on the network behaviour [3]. A nonlinear summation of the synaptic currents is introduced that incorporates local feedback from the membrane potential and an ‘effective’ time constant that varies inversely with the amount of input. The result is a more physiologically detailed description of the synaptic current produced by post-synaptic potentials. The fixed-points of the new system are found and a perturbation analysis is performed. The stability of the system is determined and a bifurcation diagram is generated using the external input and network balance as bifurcation parameters. These results are then compared to that of the original model with current-based synapses and the differences interpreted physiologically. The fixed points, dynamics and oscillatory properties of the conductance-based model differ significantly from the current-based model. This is largely due to the ‘shunting’ effect of the synapses, which acts as a network regulatory mechanism. In particular, oscillatory behaviour in the conductance-based model is suppressed. Hence, conductance-based synapses are an important physiological structure whose mechanism of synaptic transmission should not be neglected in mean-field models, particularly when applied to epilepsy.
